# Clinic Reports

**Published:** 1892-02

**Authors:** W. F. Westmoreland

**Affiliations:** Professor of Principle and Practice of Surgery, Atlanta Medical College


### CLINIC REPORTS.

### REPORT OF CLINIC OF W. F. WESTMORELAND, M. D.,

Professor of Principles and Practice of Surgery,

Atlanta Medical College.

GENTLEMEN—We present to you to-day two cases for operation, one an amputation of the forearm, the other an amputation of the breast. The history of these patients you are already acquainted with. Before beginning these operations, I wish to impress upon this class the great importance of the primary details and dressing as regards the successful progress of a case after an operation, and that by thoroughly carrying out the details of antiseptic surgery, particularly in reference to the immediate field, you can operate under the most adverse circumstances, and have primary union without any process of suppuration.

Antiseptic surgery has been surrounded by so many details, the technique that is claimed as absolutely necessary is so difficult to carry out that student, and general practitioner, is apt to consider its accomplishment beyond his ken ; that it may be achieved by surgeons in special hospitals, but impossible in the hands of the surgeon from house to house—a palace to-day, a hovel to-morrow.

There could be no worse surroundings than we have in this amphitheatre where we expect to operate to-day. The room is crowded with students all day, used by the professors of each different branch to exhibit their patients, bringing together within these walls people suffering from every imaginable disease. Less than an hour ago, a cadaver, in the last stage of dissecting, was demonstrated before you here. In addition to these general surroundings, both the cases we expect to operate upon have had long continued suppuration. We have prepared for these operations before the class, so you watch all the different stages.

A fountain syringe, filled with hot bi-chloride solution (1-2000) is hung within easy reach, four basins filled with the same solution are placed near at hand ; into the first we place half a dozen towels ; in the next, the sponges taken from a jar of carbolized solution (1-20) where they have been kept pure ; the solution in the remaining pans is used by the operator and assistants for washing their hands. The instruments are placed in a pan containing carbolized solution (1-40). Several sizes of cat-gut, which will be used as sutures and ligatures, are in alcohol in this bottle. The patients have been prepared by the parts surrounding the field of operation being thoroughly scrubbed with nail brush and soap ; the hair is shaved off; a sponge saturated with ether will remove all the oily secretions that may have found lodging around the edges of the fistulous tracts. Now wash with bi-chloride solution (1-500) and rinse with milder solution (1-2000) and put on an antiseptic dressing or towels wrung out of bi-chloride solution to keep the parts pure until time of operation.

CASE I.—*Amputation lower third forearm, for arthritis of the wrist joint.*

The patient has had long continued suppuration and the bones of the joint are now disintegrated, the distal ends of the ulna and radius being involved.

The patient is now anæsthetized, so we will proceed with the operation. My own hands and those of the assistants are thoroughly scrubbed with a nail brush and soap, then rinsed in the pan of bi-chloride solution. The patient's arm is douched with the same solution, and protected by having towels from bi-chloride solution placed around it. Esmarch's bandage for the bloodless operation is applied. We amputate by the circular flap; ligate all vessels with cat-gut before removing the bandage. The wound is now ready to be closed. This is done by bringing the edges together with a continuous cat-gut suture. Now, we come to the very important point of drainage. You see we have made an incision through the flap on the outer side and another on the under surface of the arm. Instead of using drainage tubes, which many of you will never keep, we will retrovert the edges of the incisions and stitch them back with cat-gut. This insures perfect drainage. After the cavity is washed out thoroughly with a stream of solution from the fountain syringe, the dressing is applied. This consists of a number of layers of bi-chloride gauze smoothly applied and carried as high as the elbow. This is held in position by a bandage of the same material, only slight pressure being made. The arm is now enveloped in a moderately thick layer of borated cotton, which is held by a firmly applied bandage. We now put the arm on a well padded straight split to insure perfect rest. Last of all, we put on a starch bandage, which, when dried, holds the dressings firmly in position and prevents any slipping.

CASE 2.—*Amputation of breast for non-malignant tumor, in a stage of suppurative inflammation, with numerous fistulous tracts. Axillary glands not involved.*

Patient prepared in the same manner as the previous one. The breast removed by an irregular incision so as to include the fistulous openings, wound closed by continuous cat-gut suture; an incision made at the most dependent angle of wound, edges of the incision retroverted and stitched as in previous case. Same dressings used and arm bandaged to side to give perfect rest.

When we finished dressing these cases, the fate of the wound as to whether there would or would not be suppuration was settled, as it always is, with the first dressing. I have not noticed any omission of antiseptic precautions and feel that I can say positively, that we will have primary union without any suppuration, although the surroundings are bad and the condition of the patients still worse. Occasionally during the operation a stream of solution was allowed to play on the operative field; the wound was thoroughly washed immediately before the dressing was applied. We made ample drainage, and put on a dressing that will give perfect rest. So the three essentials of antiseptic surgery, antiseptic details, perfect drainage and complete rest, were carried out.

As to the complaint that it makes a much longer operation, my best answer is that it has taken slightly over a half an hour to make these operations and dress the patients, part of which time was occupied in removing our patient and bringing in the other.

Everything we have used in these operations is easily procured, and can be carried to the bedside of your patient in a satchel.

